# Proceedings of N. Y. Homœopathic Union

**Published:** 1888-09

**Authors:** Clarence Willard Butler

**Affiliations:** Secretary


### PROCEEDINGS OF THE NEW YORK HOMŒOPATHIC UNION.

The meeting was called to order by President Bayard at 8.30 P. M., May 17th.

The minutes of the previous meeting were read and approved. Referring to Dr. Butler's suggestion that a more careful study of Pathological Anatomy, with reference to drug action, might give us valuable indications for drug application, Dr. P. P. Wells said he who prescribes from a pathological standpoint places his trust in a stool which, so far as the knowledge of the prescriber was concerned, had but one leg. The knowledge of the diseased condition is his, or often, perhaps, some one's *theory* of that condition, but where was he to find drugs which had produced the many tissue changes met in practice?

Dr. Butler thought that such tissue changes might furnish us with valuable indications therapeutically, obtained as the result of toxic doses of the drug or of clinical observations; but these would ever be secondary to the subjective symptom.

Dr. Thomson agreed with Dr. Wells, and believed Dr. Butler's position to be untenable.

The Secretary then read Section V of the *Organon* from the Stratton translation.

Dr. Eaton read the same, in comparison, from Wesselhœft's translation, and Dr. Fincke read his own translation, as follows:

"As aids to healing serve to the physician the data of the most probable *occasioning* of the acute disease, as also the most significant moments of the whole history of the chronic sickness, in order to find out the *original cause* which mostly depends upon a chronic miasm, whereby the cognizable constitution of the (especially chronic) patient, his emotional and mental character, his occupations, his mode of living and his habits, his public and domestic relations, his age and his sexual functions, etc., have to be taken into consideration."

Dr. Thomson.—Is not the chronic miasm a secondary cause, derived from our ancestors, perhaps, not a true cause?

Dr. Fincke.—One cause. The cause is "that which lies at the bottom."

Section VI reads: "The unprejudiced observer, he knows the nullity of supersensual speculations, which cannot be proven by experience, even if he be the most acute, in each single disease perceives nothing but variations of the state of body and mind, cognizable externally by the senses, *disease signs, incidents, symptoms, i. e.*, deviations from the healthy previous state of the now sick, which he himself feels, which the bystanders perceive in him, and which the physician observes on him. All these cognizable signs represent the disease in its whole compass, *i. e.*, they together constitute the true and only thinkable form of the disease. (1) Hence I do not know how it was possible, that without carefully observing the symptoms and following them in the cure, they fell upon the idea to be driven to seek and to be enabled to find for that what was to be healed in the disease, only in the obscure and indescernible interior, under the bragging and ridiculous pretention to be able to perceive the changes in the indescernible interior without paying particular attention to the symptoms, and to set them in order again with (unknown) remedies, and to call such a proceeding the only means to cure thoroughly and rationally. Is not, for the healing artist, that what by the senses is cognizable by signs in disease, the disease itself? Since he never can see the spiritual being which creates the disease, the life force, and never even need see her, but only her morbid actions in order to heal the disease accordingly. Why, besides, does the old school want to find out a *prima causa morbi* in the hidden interior, but in turn does reject and superciliously despise as objects of healing the sensually and distinctly perceivable representation of the disease, the symptoms which speak to us audibly? What else does he want to cure, if not these?"*

Dr. Wells wished to emphasize the fact that our only knowledge therapeutically of disease was its perceptible symptoms and to take exception to speaking of disease as an entity—as anything but these perceptible phenomena. "It is misleading and untrue."

Dr. Bayard.—Hahnemann was a simple vitalist. Harmony in vital action is health—variation in vital harmonies is disease.

Section VII was next read, Dr. Fincke's translation :

"Since, then, in disease, from which no evidently occasioning and sustaining cause (*causa occasionalis*) is to be removed. (1) Nothing else can be perceived than the disease signs, therefore, with regard to possible miasm and under observation of the concomitant circumstances (Sec. V), it must be only the symptom by which the disease claims the medicine suited for its help and by which it is able to point it out, therefore, the complex of these her symptoms, *this picture, reflecting outwardly the internal essence of the disease, i. e., the suffering of the life force,* must be the main and only object by which the disease can indicate the remedy needed—the only point which can decide the selection of the most appropriate remedy—therefore, in one word, the complex of the symptoms. (2) For the healing artist, must be the main and only point which in every case of disease he has to discern and which he has to *take away* by his art, so that it be healed and converted into health.

"(1) It is self-understood that every sensible physician will

*The physician, searching for the hidden relations in the interior of the organism can err daily; but the homœopathician, in taking up the total group of symptoms with the necessary care, has a true guide, and if he succeeds in removing the whole group of symptoms, he surely has also removed the interior hidden cause of the disease.—Rau. l. c. p. 103.

remove these at first, then the indisposition usually ceases by itself. He will remove from the room the strong-smelling flowers which excite fainting and hysterical conditions, he will draw the splinter out of the cornea which excites ophthalmia, he will loosen the too tight bandage threatening mortification and put it on more suitably, he will lay bare and tie up the wounded artery which induces syncope, he will try to expell the swallowed berries of Belladonna, etc., by vomiting, he will extract the foreign bodies which have come in the openings of the body (nose, pharynx, ears, urethra, rectum, vagina), he will crush the stone in the bladder, he will open the imperforate anus of the new-born child, etc.

"(2) At all times the old school in diseases has tried to combat or if possible to suppress a single one of the many symptoms of medicine, a *onesidness*, which under the name, *symptomatic method of cure*, has justly excited universal contempt, because not only nothing is gained by it, but also much is spoiled. A single one of the present symptoms is as little the disease itself as a single foot is the man himself. This method was the more objectionable since they treated such a single symptom only by an opposite remedy (hence, only enantiopathically and palliatively), and thereby after a short relief is afterward much more aggravated."

Dr. Bayard—That note is sound; a palliative is a cloak.

Dr. Wells—Yes, and the cloak don't cover the palliative, don't palliate.

Dr. Fincke next presented a paper on "Hahnemann's Life Force." This paper, which showed great diligence of research and intimate acquaintance with "The Master's" writings, was received with applause.

Dr. Butler innocently remarked that he didn't know what a "spirit" was. This precipitated a long metaphysical discussion which your Secretary acknowledges himself wholly unable to report.

Dr. Carlton called attention to Hahnemann's note to Section VII regarding single symptoms as a basis for a prescription. He thought it evident that Hahnemann did not approve of prescribing upon the single symptom, even though a characteristic —a "key-note."

Dr. Wells—Such prescribing will sometimes cure acute, but never chronic cases.

Dr. Fincke wished to know if, while treating a chronic case an acute disease, *e. g.*, malaria, should develop, he should turn to the treatment of the acute trouble and ignore the chronic disease?

Dr. Wells—*No*, look at your patient anew, get, if possible, the remedy which covers the symptoms of both the acute and the chronic disease (and such drug can almost always be found); give it in a low potency, so Dr. Hering advised, and you will cure both diseases—your patient, in other words.

Dr. Smith asked why the low potency was to be chosen.

Dr. Wells—That was Hering's advice, those were his views when I conversed with him on this subject more than fifty years ago. Whether he modified them in later years I do not know.

Dr. Bayard said that many of us had modified the views of our younger years. He thought Dr. Hering had done so in this respect.

Dr. Miller said that his experience was not in accord with such recommendation.

Dr. Bayard advised "prescribe at the right time when possible, and in the right direction. If, for example, your patient presents a history of syphilis be sure and recognize that as a factor in the case."

On account of the I. H. A. meeting in June, adjournment was had, on motion of Dr. Carlton, for two months.

Present, Drs. Eaton, Finch, Thomson, Close, Van Evera, S. N. Smith, Cameron, Spottiswoode, Myers, Carlton, Williams, Knudsen, Howard, Bedell, Deschere, Palmer, P. P. Wells, E. Bayard, B. Fincke.

Clarence Willard Butler, *Secretary*.

